# Calibrating the Severity of Forest Defoliation by Pine Processionary Moth with Landsat and UAV Imagery

**DOI:** 10.3390/s18103278

**Published:** 2018-09-29

**Authors:** Kaori Otsu, Magda Pla, Jordi Vayreda, Lluís Brotons

**Affiliations:** 1 Centre for Ecological Research and Forestry Applications (CREAF), 08193 Cerdanyola del Vallès, Spain; j.vayreda@creaf.uab.cat; 2 InForest JRU (CTFC-CREAF), 25280 Solsona, Spain; magda.pla@ctfc.cat; 3 Spanish National Research Council (CSIC), 08193 Cerdanyola del Vallès, Spain

**Keywords:** forest defoliation, *Thaumetopoea pityocampa*, vegetation index, unmanned aerial vehicle (UAV), change detection

## Abstract

The pine processionary moth (*Thaumetopoea pityocampa* Dennis and Schiff.), one of the major defoliating insects in Mediterranean forests, has become an increasing threat to the forest health of the region over the past two decades. After a recent outbreak of *T. pityocampa* in Catalonia, Spain, we attempted to estimate the damage severity by capturing the maximum defoliation period over winter between pre-outbreak and post-outbreak images. The difference in vegetation index (dVI) derived from Landsat 8 was used as the change detection indicator and was further calibrated with Unmanned Aerial Vehicle (UAV) imagery. Regression models between predicted dVIs and observed defoliation degrees by UAV were compared among five selected dVIs for the coefficient of determination. Our results found the highest R-squared value (0.815) using Moisture Stress Index (MSI), with an overall accuracy of 72%, as a promising approach for estimating the severity of defoliation in affected areas where ground-truth data is limited. We concluded with the high potential of using UAVs as an alternative method to obtain ground-truth data for cost-effectively monitoring forest health. In future studies, combining UAV images with satellite data may be considered to validate model predictions of the forest condition for developing ecosystem service tools.

## 1. Introduction

Currently, pest insects are the principal biotic drivers causing disturbances threatening Mediterranean forests in combination with abiotic factors such as drought, fire and climate change [1,2]. The pine processionary moth (*Thaumetopoea pityocampa* Dennis and Schiff.), one of the major defoliating insects in Mediterranean pine forests, has been considered by the Intergovernmental Panel on Climate Change (IPCC) as an indicator of global warming [3] for being recorded in an expanded biogeographical range of host distribution from Southern Europe in the Mediterranean region towards northern latitudes and higher elevations over the past twenty years [4,5]. Consequently, the defoliators may continue to increase with the future trend of climate change scenarios [5].

Outbreaks of *T. pityocampa* have been observed to be cycles of 6 years on average [2,6], mainly in managed young stands [5,7]. In a normal year healthy forest stands recover from defoliation, however, periodic outbreaks attack large forests in which host trees may suffer up to 100% defoliation. Thus, severely affected stands may result in significant reductions of individual tree growth, stand productivity, and forest ecosystem health, from tree to stand and landscape levels [5,8]. Such growth reduction may accelerate the rate of tree mortality when the damage is accumulated due to other biotic factors as well as abiotic factors [9]. As a consequence, defoliation focused on host pine trees is more likely to change the structure and species composition in natural stands as well as lose economic values in planted stands [2,9]. Furthermore, forest ecosystems at the landscape level are major carbon sinks contributing to mitigate the impacts of climate change [10,11,12]. Therefore, to predict future scenarios on forest productivity and mortality in cases where the outbreaks may occur, reliable data from monitoring the pest distribution at regional and national scales are required [5,12].

Despite the annual ground survey on forest health in the Mediterranean countries such as France, Portugal and Spain which are frequently affected by outbreaks of *T. pityocampa*, we currently lack accurate, fine-grained and timely information systems for monitoring the latest forest condition. Thus, an efficient tool to improve such regional or national monitoring systems spatially and temporally may be developed by adequately using the latest remote sensing technologies. In addition to conventional field surveys which are often time-consuming to cover large areas, satellite-based images such as MODIS and Landsat at medium spatial resolution (30–250 m) have been widely used for detecting defoliations in forest systems over the past two decades [1]. Currently, due to the free accessibility to medium-resolution (10–60 m) products from Landsat 8 [13] and Sentinel-2 [14], they are most commonly used for cost-effectively monitoring large areas. For sensors at high spatial resolution (<10 m), the private industry continued to launch satellites such as IKONOS, QuickBird, RapidEye and TerraSAR-X [1]. With further advancements in spaceborne technology, the sensor’s spatial resolution nowadays can be as high as 0.3 m (WorldView-4), and continue to enhance temporal and spectral resolution as well [15]. In Spain, using various sensors (Airborne Hyperspectral Scanner, Hyperion, ChrisProba, Quickbird, and Landsat), Cabello et al. [16] have applied vegetation indices for mapping forest damage caused by *T. pityocampa* to estimate the Leaf Area Index in affected pine stands, followed by Sangüesa-Barreda et al. [2] with the combined method of Landsat-derived vegetation indices and dendrochronology for assessing the tree growth reduction affected by outbreaks of *T. pityocampa.* Furthermore, the emergence of airborne laser scanning (ALS) characterized by point clouds complements the three-dimensional (3D) structure in addition to the spatial resolution higher than any spaceborne technology [1,17]. Using ALS metrics, classification of defoliated Scots pines at the individual tree level was demonstrated by Kantola et al. [18].

Since the cost of above high-resolution products remains a limiting factor for small operational areas, the trend of monitoring forest health in recent studies has shifted to the alternative 3D technology based on cost-effective Unmanned Aerial Vehicle (UAV) at high temporal and spatial resolution over the past decade [19]. While initial studies with UAVs were focused on crop management for agriculture applications, the latest UAV technology has proved to be effective for forestry applications (forest disturbances and diseases, forest cover mapping, tree species identification, and forest inventory measurements) as a sampling tool for acquiring ground-truth data [20]. To date only a few studies have examined on the classification accuracy of forest insect defoliations using UAV imagery. The classification methods include Random Forest [19], object-based image analysis (OBIA) [21,22], k-Nearest Neighbor [23], and maximum likelihood [24,25]. Moreover, no study has calibrated satellite-based vegetation indices as a predictive indicator of such defoliation with UAV-derived data as ground-truth while Pla et al. [26] has recently made progress in calibrating some indices specific to fire damage using UAV imagery.

With the objective to quantify forest response to infestation, we developed remote sensing-derived indicators to measure the defoliation levels. In this study, we aim to evaluate the spatiotemporal degree of defoliation during a recent outbreak of *T. pityocampa* in Mediterranean pine forests by change detection analysis using a combination of satellite and UAV imagery. Our main objectives are: (1) examine regression models between vegetation indices (VI) derived from Landsat imagery and defoliation degrees interpreted by UAV imagery for calibration; (2) map defoliation classes based on the best-fit VI model to assess classification accuracy for validation.

## 2. Materials and Methods

### 2.1. Study Area

**Figure 1:**
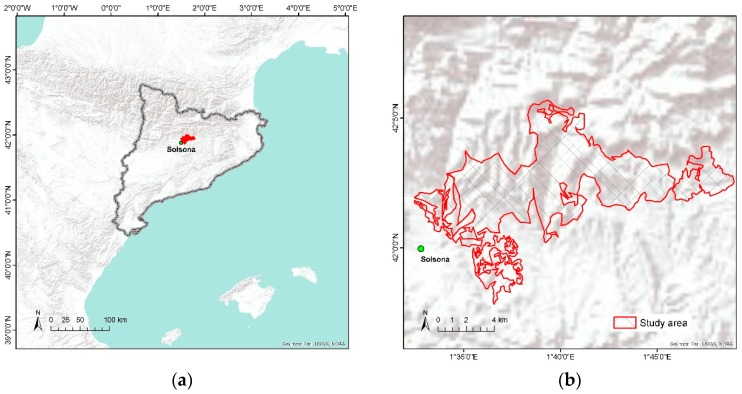
Study area showing: (**a**) Location of Solsona (41°59′40″ N, 1°31′04″ E), Catalonia (solid gray line), Spain; (**b**) one of the most severely affected areas mapped by rural agents (2016).

The study area is located near the city of Solsona, Catalonia, northeast of Spain (Figure 1a), encompassing 6809 hectares (Figure 1b) dominated by pine forests (elevation above sea level ranging from 600 m to 1100 m). Primary host species are *Pinus nigra* followed by *P. sylvestris*, which are the dominant tree species in mixed stands with *Quercus ilex* and *Q. humilis*. The climate is Mediterranean continental characterized by hot summer and cold winter. According to the local meteorological station, the mean annual temperature is 11.7 °C and the mean total annual precipitation is 864 mm. As larvae of *T. pityocampa* develop across winter, it has benefited from recent warmer fall and winter temperatures for overcoming the thermal thresholds and consequently increasing the rate of larval survival and growth as far as the host trees are present [5].

### 2.2. Field Data

Ground and aerial sketch mapping data were first obtained from the regional forest health inventory during the years from 2010 to 2016. Since the project on evaluating the severity of infestations caused by *T. pityocampa* started, field surveys have been conducted by rural agents every winter when the symptoms are visually most evident from February to March after the larvae complete feeding on needles and go into the soil for pupation [4]. The survey form is standardized by the government (Generalitat de Catalunya) and filled out by rural agents. Affected stands are mapped as polygons, on average about 137 hectares per polygon ranging to the smallest of 0.03 hectare, with attributes such as observation date, forest type, aspect, tree species, and infestation severity. The severity scales are ranked from 1 to 4 at highest as follows: (1) several nests at the margins and some nests in the center of the forest stand; (2) partial defoliation at the margins on some trees and several nests in the center of the forest stand; (3) intense defoliation at the margins and partial defoliation in the forest stand; (4) very intense defoliation both at the margins and in the center of the forest stand. The total area infested by *T. pityocampa* was recorded at largest in 2016. Based on this inventory database with sketch map polygons, our study area was selected to be one of the most severely affected areas during winter 2015–2016 (Figure 1b).

### 2.3. Landsat Images and Vegetation Indices

Pre-outbreak (20 September 2015) and post-outbreak (30 March 2016) images were obtained from Landsat 8 (path 198, row 31) with minimum clouds to capture the maximum defoliation period from fall to spring. Surface Reflectance (SR) products were downloaded from the GloVis (http://glovis.usgs.gov) USGS server. Furthermore, the Band Quality Assessment included in the SR product was applied to exclude pixels described as water, snow, cloud, and cloud shadow so that only pixels with values indicating clear terrain were extracted [2,26,27]. Preprocessed images in the cartographic UTM projection were further extracted by forest cover classified as pine-dominated stands according to the Land Cover Map of Catalonia (MCSC) generated in 2009. The best spectral regions for defoliation detection may correspond to reflectance bands from Landsat 8 such as near infrared (NIR = Band 5) and shortwave infrared (SWIR1 = Band 6 and SWIR2 = Band 7) which enable to capture a fast reduction of pine foliage and the consequent reduction in tree evapotranspiration [1,26]. In this study, we tested five VIs to predict the defoliation degree in pine forests as summarized in Table 1.

Comparing values of VI among multiple dates may function as a good indicator for change detection [1,4,16,17,18]. To distinguish annual defoliation during the outbreak from cumulative defoliation over several years, a pre-outbreak (20 September 2015) image was compared to additional images from Landsat 8 representing the month of September in the previous two years (2013 and 2014) to confirm that there was no abrupt change in the VI values used in our study area before outbreak. The difference in VI (dVI) was calculated by simply subtracting the value of each VI in year 2016 (post-outbreak) from year 2015 (pre-outbreak) to estimate annual defoliation:

VI (pre-outbreak) − VI (post-outbreak) = dVI(1)

### 2.4. Visual Interpretation with UAV Images

**Figure 2:**
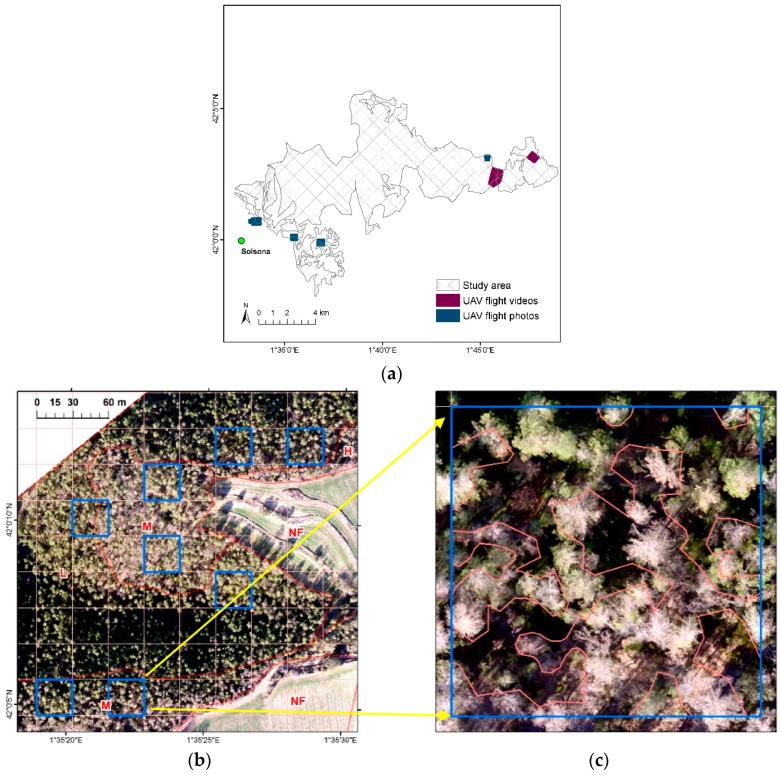
Observed defoliations based on UAV imagery showing: (**a**) the location of UAV sample images captured in photos and videos where sketch map polygons were identified as most severely affected areas in 2016; (**b**) an example of the orthomosaic stratified by land cover and defoliation degree (NF—not forested, L—low, M—medium, H—high) showing selected grid cells of 30 m × 30 m; (**c**) visual interpretation of defoliation in percentage per grid cell (50% in this sample).

To assess the level of defoliation in selected locations, we used a camera (FC200, DJI, Shenzhen, China) mounted on a UAV (Phantom 2 vision+, DJI, Shenzhen, China). This camera can capture images, photos or videos, in the visible spectrum (RGB including blue, green and red bands) with a lens focal length of 5 mm and field of view of 120 degrees [33]. The RGB imagery had a 14 megapixels-resolution and was collected from a flying altitude ranging at 50–100 m above the ground level, resulting in a ground sample distance (GSD) of 2.0–3.5 cm. Seven flight surveys were conducted between December 2015 and March 2016 covering approximately 193 hectares across the study area shown in Figure 2a, of which five were collected in photos (81 hectares) and two were recorded in videos (112 hectares) at speeds ranging from 4–8 m per second depending on light conditions. The survey locations were selected to represent the stand types characterized by *P. nigra* and *P. sylvestris* and by various defoliation levels. Some surveys were recorded earlier than the post-outbreak image (30 March 2016) where high defoliation levels had been already observed at that time.

The total of 526 adjacent photos overlapped (forward 80%, side 60%) from the five flights with their geotagged locations were processed in the software PhotoScan Professional 1.4.0 (Agisoft LLC, St. Petersburg, Russia). In the image processing they were geometrically aligned to build a point cloud, 3D model, digital elevation model (DEM), and finally five orthomosaic images [34]. To collect more sample images, we included additional two videos flying at 4 m per second and recording 29 frames per second with the associated GPS files, which were imported into the software Video GeoTagger (Remote GeoSystems, Fort Collins, CO, USA) to tag geolocations. Extracting a frame per second resulted in the total of 339 frames in photo files. We repeated the same image processing used for the photos to generate two additional orthomosaic images derived from the video frames.

Using the ArcGIS version 10.5 software (ESRI, Redlands, CA, USA) each orthomosaic was georeferenced to the Landsat imagery in the UTM projection by performing a first order polynomial transformation with 4 control points, obtaining an accuracy of sub-meter root mean square error. The georeferenced orthomosaic images were further stratified to delineate non-forested or forested areas with the severity of defoliation in homogenously affected stands by visual interpretation (Figure 2b).

As summarized in Table 2, the sampled grid cells of 30 m × 30 m on orthomosaic images, corresponding to the pixels captured by Landsat, were haphazardly selected by an analyst avoiding shadow pixels from each representative category. Upon delineating the surface area containing defoliated trees per grid cell, the defoliated portion of the total cell area (900 m^2^) was calculated and expressed at 5% interval (Figure 2c). Finally, all selected grid cells were classified into four categories: nil (no change = 0–5%); low (defoliation 10–30%); medium (defoliation 35–65%); and high (defoliation 70–100%), as a modified classification suggested by Hall et al. [35].

### 2.5. Regression Analysis and Threshold Classification

We statistically analyzed relationships between two variables, predicted dVI (*X*) from Landsat imagery and observed defoliation % (*Y*) from UAV imagery. The relationships between *X* and *Y* were evaluated using the software R in various regression models including simple linear, logarithmic, exponential, polynomial, and logistic models. Provided that the range of defoliation % (*Y*) is limited as the response variable is bounded from 0 to 1 [36,37,38], logistic regression was employed to express proportion defoliated, following the equation:

(2) $$Y=\frac{1}{1+e^{-\left(a+bX\right)}}$$

where *X* represents the value of pixel in a selected dVI within the sample image, *Y* represents the percentage of defoliation interpreted within an individual UAV sample image, and *a* and *b* are the slope and intercept of the regression function.

Based on fifty selected samples (sample size = 50) in the study area, regression models were fitted to evaluate the coefficient of determination, McFadden’s R^2^ [39], which is most often reported in statistical software and recommended for measures of fit for logistic regression [40]. The dVI models were then compared to determine the best-fit indicator for estimating the severity of defoliation. With the known *Y* values of 10, 35 and 70 to separate the range of defoliation %, threshold limits in dVI (*X*) from the Equation (2) were recalculated as follows:

(3) $$X=\frac{ln\left(\frac{Y}{1-Y}\right)-a}{b}$$

Finally, as defined in Table 2, the severity map of defoliation was generated by threshold classification [26,41,42].

### 2.6. Classification Accuracy

To assess an overall accuracy of the threshold classification, we generated a confusion matrix of the four defoliation classes in the 50 selected grid cells, which were compared between the Landsat-based prediction and UAV-based interpretation. Validation of classes in the severity map was performed by assessing classification accuracy of dVIs referenced to UAV observations.

## 3. Results

**Figure 3:**
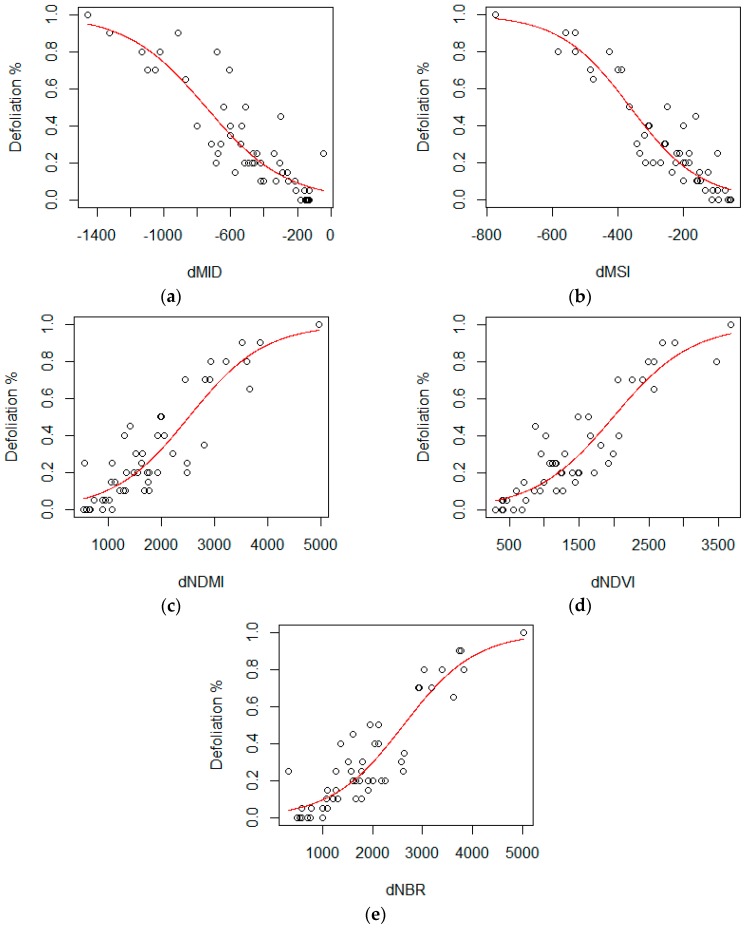
Scatterplots of defoliation (%) as response variable and dVI as predictor variable: (**a**) dMID; (**b**) dMSI; (**c**) dNDMI; (**d**) dNDVI; (**e**) dNBR.

In Figure 3 the resulting X and Y points were plotted and compared among selected VIs. Moreover, the coefficient of determination (R^2^) by dVI is summarized in Table 3, which was statistically analyzed for logistic regression proposed by McFadden [39]. The goodness of fit was highest = 0.815 with dMSI while it was not improved by normalizing VIs such as dNDMI, dNDVI, and dNBR.

**Figure 4:**
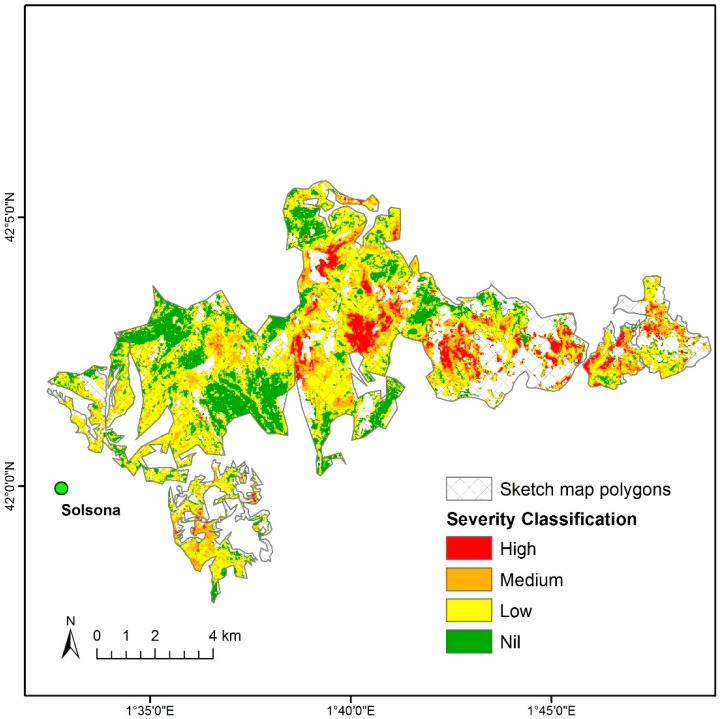
Severity map of defoliation classified by threshold limits of dMSI representing pine-dominant stands (excluding non-forested areas and stands dominated by other tree species).

Using the equations in Table 3, the range of dVI values were determined as threshold limits in classification (Table 4). Applying values in dMSI to defoliation %, any value higher than −125 is classified as no defoliation (<10%): −125 to −295 (10–35%); −295 to −453 (35–70%); and lower than −453 (>70%). Based on the threshold limits, pixels assigned to four classes of defoliation were mapped to show the severity across the study area (Figure 4). The blank pixels in no color indicate either non-forested areas or stands dominated by other tree species, which were initially excluded from the analysis.

The overall accuracy of the threshold classification was presented in Table 5 for the four defoliation classes (nil, low, medium, and high) in the 50 selected samples of dMSI referenced to UAV orthomosaic images. Greatest producer’s accuracy representing a measure of omission error was 90% for the nil defoliation class, where nine out of the 10 cells observed as nil were correctly classified by predicted dMSI. On the other hand, the high defoliation class was mapped with greatest user’s accuracy of 86% indicating commission error, which six out the seven cells predicted as high correctly represented the observed class. Overall, it should be noted that the number of cells classified as nil or medium defoliation was overestimated whereas the one classified as low or high defoliation was underestimated. Finally, the overall accuracy of the classification was 72% calculated by the ratio between the sum of the cells correctly classified from each class and 50 cells in total.

## 4. Discussion

Among five VIs tested in our study, the results with dMSI as the best predictor were consistent with recent studies on insect defoliations [2,36,37,38]. The SWIR band calculated in dMSI is known to be a good indicator for the plant moisture content in addition to the plant stress detected in the NIR band [1,43]. It may be assumed that the MSI better indicated early symptoms of the host trees stressed by dehydration in our study. The first study examining MSI in relation to conifer damage was conducted by Vogelmann et al. [44], resulted with an R^2^ of 0.830 in linear regression. Other studies with MSI have been conducted in pine forests by Sangüesa-Barreda et al. [2] demonstrating the highest significance on ANOVA tests and most recently by Zhu et al. [38] with an R^2^ of 0.982 in logistic regression. The MSI was also effectively applied to defoliation in deciduous forests by Townsend et al. [36] with an R^2^ of 0.844 in logistic regression and Rullán-Silva et al. [37] with an R^2^ of 0.632 in sigmoidal mixed-effects models. For monitoring coniferous forests in general, the MSI has been found to be more effective than the NDVI which has been mainly applied to deciduous forests [38,43,44]. Nevertheless, several potentially robust VIs should be tested on each particular study area since tree-insect relationships vary from site to site [1].

Our initial attempt was to use sketch map polygons from field data as training samples for supervised classification on the severity of defoliation. However, the significant discrepancy in spatial resolution between the field data provided by regional rural agents and Landsat data became evident. The sketch map polygons were delineated for classifying severity levels at a coarse scale in hectares including non-forested areas whereas the spatial resolution of Landsat imagery is as fine as 30 m per pixel, which resulted in a wide range of values among pixels within the same polygon. Yet, without any ground observation such as nests of *T. pityocampa*, it is often difficult to distinguish the cause of defoliation based on only spectral bands or even aerial surveys at low levels of defoliation [8]. Further integration by training the rural agents to apply UAV workflows to their annual health survey may fill this monitoring gap. As suggested in the most recent review on forest health monitoring by Hall et al. [35], how spaceborne and airborne remote sensing may be integrated with aerial and field surveys into a multi-scale, multi-source monitoring system should be explored.

Regarding stand dynamics and species compositions, we extracted pine-dominated stands from the Land Cover Map of Catalonia (MCSC) and assumed that sampled stands were dense enough to represent Landsat VIs based on the defoliation degree of dominant species in pine stands. Nonetheless, we acknowledge the possibility of misrepresenting the VIs in severely defoliated stands to some extent where understory species is non-host evergreen such as *Q. ilex* which is not affected by *T. pityocampa*. In such cases, healthy understory trees below defoliated pine trees may have reflected more greenness at the stand level. Moreover, it is also possible to overestimate the defoliation degree in stands mixed with non-host deciduous trees shedding their leaves in winter [35]. This issue of overestimating or underestimating the impacts on host trees can be minimized by discrimination of deciduous species by detecting spectral variations due to vegetation phenology with Landsat time-series approach such as LandTrendr which can monitor cumulative defoliation as well as annual defoliation [45] while the satellite-based spatial resolution is not high enough to identify individual trees. Thus, for species identification at the tree level recent studies with UAV-derived multispectral bands and the associated indices [20] may be further investigated to discriminate only those species of interest for calibration and validation of defoliation degrees.

Regression analysis may be improved by increasing the sample size in each severity category or the number of predictive parameters, or testing transformed dVIs [26,41,42]. One simple way to increase the sample size can be achieved by reducing the cell size for sampling VIs from 30 m in Landsat 8 to 20 m in Sentinel-2 imagery. As multiple predictive parameters climate data (temperature and precipitation) or topographic features (elevation, slope and orientation) may be considered to improve the coefficient of determination in regression models. Moreover, we may introduce the number of nests formed by *T. pityocampa* as an additional predictive parameter to estimate the infestation severity as recent studies with the UAV technology have attempted to examine the severity of infestation at the individual branch level [24,25]. However, the number of nests captured by UAV images from the air may be potentially underestimated if some nests on lower branches or in dense stands are not counted. Regarding observed parameters, our assessment on UAV-derived defoliation levels (%) was limited to manual photointerpretation, including shadows where some uncertainty remains. The automated removal of shadow pixels should be explored in future studies by testing various thresholds on spectral bands.

Threshold classification based on regression models in this study was one method to generate the defoliation severity map with the advantage of increasing or decreasing the number of classes by changing threshold limits of dVI (X) corresponding to the continuous defoliation degree (Y). Other classification methods using non-parametric algorithms may be taken into further consideration with a larger sample size to find the optimal method among unsupervised (ISODATA, K-means), supervised (maximum likelihood), and machine learning such as Random Forest, Decision Trees, k-Nearest Neighbor, and Support Vector Machine [46]. In general, those studies based on non-parametric models demonstrated that the classification accuracy significantly increased when the number of classes decreased [20,23].

Tradeoffs between spatial, temporal, and spectral resolution are critical to determine classification specific to case study. In our study sketch map polygons served as the primary information to filter affected areas most likely by *T. pityocampa* despite low spatial and temporal resolution. The defoliations over winter due to *T. pityocampa* can be discriminated by seasonal activity from other potential causes for defoliations during summer such as drought and summer-feeding insects [38]. Satellite-based Landsat imagery, at medium spatial and temporal resolution, enabled us to calculate various dVIs due to high spectral resolution. While Landsat has the advantage of allowing time-series analysis from the data archive to track back to 1972 [13], the use of Sentinel-2 has been recently increasing since its launch in 2015 due to the public access available at the higher temporal, spatial and spectral resolution (every 5 days at 10 m, 20 m, or 60 m for 13 bands) [14] than that of Landsat 8 (every 16 days at 15 m, 30 m, or 100 m for 11 bands). As shown in Figure 4, those dVIs at medium spatial resolution need to be calibrated with field observations to estimate the severity classification at a regional scale. Thus UAV imagery observed at high spatial and temporal resolution may improve the efficiency of such calibration. Yet, current limitations of UAV technology include battery duration for 20–30 min, associated small area coverage for sample images per flight, and imagery acquisition permission due to privacy issues specific to some countries [20,21]. To cover a large area at landscape and regional scales, it would require multiple flights that may not be consistent with the time, sensor and weather conditions, therefore, it would not replace satellite-based imagery.

Nonetheless, the latest UAV can obtain dense point clouds and multispectral bands (sensors for red edge, NIR and SWIR outside the visible spectrum), which may be a promising technology with a high spatial and spectral resolution for small-scale forestry applications. Using the density of points at a tree level, as successfully demonstrated by Näsi et al. [23], the structural change in individual trees may be detected and monitored for cumulative defoliation. While some studies [23,47,48,49] have used the UAV-derived NDVI as the most robust indicator for their analysis on insect defoliations, future studies shall compare it to the UAV-derived MSI which can be calculated from SWIR in the latest sensor technology. Where high spectral resolution is not required for small operational areas, compared to using both satellite and UAV imagery, preparing a UAV flight would greatly increase the time-efficiency and cost-effectiveness as well as flexibility in planning imagery acquisition [19,21,23]. In addition to such advantages as alternative methods for generating orthomosaic images and calculating VIs from multispectral bands, the use of UAVs enables to avoid clouds during flights, which often cannot be controlled by satellite orbit scheduling [19,23]. Thus, with relatively less efforts and lower costs, UAV imagery may increase the spatial quality to be equivalent to ground-truth data.

## 5. Conclusions

In this study the difference in vegetation index between pre-outbreak and post-outbreak images derived from Landsat imagery was calculated for estimating the severity of defoliation. Although satellite data was calibrated with the limited number of UAV images including photos and videos available from the study area, dMSI among five satellite-derived vegetation indices resulted in the best-fit logistic regression model with an acceptable overall accuracy of 72% for the severity classification. Therefore, the use of UAV images may hold great potential as an alternative cost-effective method to other conventional ground-truth data. In future studies, new additional UAV images should be incorporated to validate previously calibrated results in the same study area or adjacent areas affected by *T. pityocampa*. Upon validation the best-fit dVI model may become a robust tool to estimate the severity of defoliation for areas where ground-truth data is limited.

## Figures and Tables

**Table 1:** Vegetation indices derived from Landsat 8 multispectral bands.

Index	Acronym	Formula	Reference
Middle Infrared Wavelengths	MID	Band 6 + Band 7	[28]
Moisture Stress Index	MSI	$\frac{\mathrm{Band}\;6}{\mathrm{Band}\;5}$	[29]
Normalized Difference Moisture Index	NDMI	$\frac{\mathrm{Band}\;5\;-\;\mathrm{Band}\;6}{\mathrm{Band}\;5\;+\;\mathrm{Band}\;6}$	[30]
Normalized Difference Vegetation Index	NDVI	$\frac{\mathrm{Band}\;5\;-\;\mathrm{Band}\;4}{\mathrm{Band}\;5\;+\;\mathrm{Band}\;4}$	[31]
Normalized Burn Ratio	NBR	$\frac{\mathrm{Band}\;5\;-\;\mathrm{Band}\;7}{\mathrm{Band}\;5\;+\;\mathrm{Band}\;7}$	[32]

**Table 2:** Selected UAV sample images for calibration in each category of defoliation severity.

Severity	Defoliation (%)	Number of Samples
Nil	0–5	10
Low	10–30	23
Medium	35–65	8
High	70–100	9

**Table 3:** Summary of logistic regression models.

Index	Equation	R^2^ (McFadden’s)
dMID	$Y=\frac{1}{1+e^{-\left(-3.1299111-0.0041928X\right)}}$	0.740
dMSI	$Y=\frac{1}{1+e^{-\left(-3.3570352-0.0092755X\right)}}$	0.815
dNDMI	$Y=\frac{1}{1+e^{-\left(-3.5552389+0.0014107X\right)}}$	0.749
dNDVI	$Y=\frac{1}{1+e^{-\left(-3.509468+0.001767X\right)}}$	0.787
dNBR	$Y=\frac{1}{1+e^{-\left(-3.6323329-0.0013874X\right)}}$	0.776

**Table 4:** Threshold limits and the range of vegetation indices.

Index	Defoliation (%)		
	10	35	70
dMID	−222	−599	−949
dMSI	−125	−295	−453
dNDMI	963	2081	3121
dNDVI	743	1636	2466
dNBR	1034	2172	3229

**Table 5:** Confusion matrix of a threshold classification using 50 pixel values of dMSI predicted from Landsat 8 in reference to four classes observed from UAV.

Class		Predicted (Landsat 8)					
		Nil	Low	Medium	High	Total	Producer’s Accuracy
**Observed (UAV)**	Nil	**9**	1	0	0	10	0.90
	Low	2	**17**	4	0	23	0.74
	Medium	0	3	**4**	1	8	0.50
	High	0	0	3	**6**	9	0.67
	Total	11	21	11	7	**50**	
	User’s Accuracy	0.82	0.81	0.36	0.86		**0.72**
